# Synthesis of Glycyrrhetinic Acid-Modified Chitosan 5-Fluorouracil Nanoparticles and Its Inhibition of Liver Cancer Characteristics *in Vitro* and *in Vivo*

**DOI:** 10.3390/md11093517

**Published:** 2013-09-17

**Authors:** Mingrong Cheng, Xiaoyan Gao, Yong Wang, Houxiang Chen, Bing He, Hongzhi Xu, Yingchun Li, Jiang Han, Zhiping Zhang

**Affiliations:** 1 Department of General Surgery, Pudong New Area District Zhoupu Hospital, Shanghai 201318, China; E-Mails: cmrlq@126.com (M.C.); hanjiang637@yahoo.com.cn (J.H.); kaiment1@126.com (Z.Z.); 2 Department of Endoscopy, Pudong New Area District Zhoupu Hospital, Shanghai 201318, China; E-Mail: 18121216087@163.com; 3 Department of Plastic Surgery, Pudong New Area District Zhoupu Hospital, Shanghai 201318, China; E-Mail:gxyhelen@126.com; 4 School of Materials Science and Engineering, Wuhan University of Technology, Wuhan 430070, China; 5 Zhejiang Huafon Fiber Research Institute, Zhejiang Huafon Spandex Co., Ltd, Wenzhou 325200, China; E-Mail: chenhouxiang1987@163.com; 6 Department of General Surgery, Shanghai Fifth People’s Hospital, Fudan University, Shanghai 200240, China; E-Mail: xuhongzhi1980@126.com

**Keywords:** hepatic carcinoma, regulatory T-cells, glycyrrhetinic acid, targeted therapy, 5-fluorouracil

## Abstract

Nanoparticle drug delivery (NDDS) is a novel system in which the drugs are delivered to the site of action by small particles in the nanometer range. Natural or synthetic polymers are used as vectors in NDDS, as they provide targeted, sustained release and biodegradability. Here, we used the chitosan and hepatoma cell-specific binding molecule, glycyrrhetinic acid (GA), to synthesize glycyrrhetinic acid-modified chitosan (GA-CTS). The synthetic product was confirmed by Fourier transformed infrared spectroscopy (FT-IR) and ^1^H-nuclear magnetic resonance (^1^H-NMR). By combining GA-CTS and 5-FU (5-fluorouracil), we obtained a GA-CTS/5-FU nanoparticle, with a particle size of 217.2 nm, a drug loading of 1.56% and a polydispersity index of 0.003. The GA-CTS/5-FU nanoparticle provided a sustained release system comprising three distinct phases of quick, steady and slow release. We demonstrated that the nanoparticle accumulated in the liver. *In vitro* data indicated that it had a dose- and time-dependent anti-cancer effect. The effective drug exposure time against hepatic cancer cells was increased in comparison with that observed with 5-FU. Additionally, GA-CTS/5-FU significantly inhibited the growth of drug-resistant hepatoma, which may compensate for the drug-resistance of 5-FU. *In vivo* studies on an orthotropic liver cancer mouse model demonstrated that GA-CTS/5-FU significantly inhibited tumor growth, resulting in increased survival time.

## 1. Introduction

5-fluorouracil (5-FU) is a first-line anticancer drug that inhibits tumor cell proliferation by interfering with the synthesis of nucleic acid. However, its efficacy is affected by low lipophilicity and low bioavailability [1,2]. In addition, its clinical use is limited by unwanted side effects, such as gastrointestinal reactions, myelosuppression, alopecia and ataxia, and by its narrow therapeutic index (the therapeutic dose is close to the toxic dose) [3]. Structural optimization of 5-FU has been undertaken in an attempt to improve selectivity and to reduce side effects. This has resulted in the discovery of a series of prodrugs associated with fewer side effects and a higher chemotherapeutic index than 5-FU (e.g., fluorouridine and tegafur, which are in clinical trials) [4,5]. However, the prodrugs are still limited by adverse effects, resulting from a lack of selectivity for cancer cells.

Drug delivery systems carry drugs to the targeted cells by exploiting the different physiological and biochemical characteristics of tumor and normal cells [6]. These systems can be used to reduce the distribution and metabolism of 5-FU in non-target organs and tissues. They also improve the drug efficacy and reduce side effects as a result of the lower doses that are administered. The natural polymer, chitosan (CTS), and its analogues have been widely studied as drug vectors, based on their lack of toxicity, biodegradability, good biocompatibility and absorption [7,8,9]. Small-molecule drugs, such as 5-FU and paclitaxel, carried by CTS or its derivatives, result in extended release, improved bioavailability and reduced side effects [10,11,12]. These carrier agents also have adhesion and biodegradability properties, which give them the potential to improve drug efficacy. Targeted drug delivery has been shown to concentrate the drugs at the site of diseased tissue, thereby greatly reducing side effects in normal tissues and improving the biodegradability and drug efficacy [13,14]. Glycyrrhetinic acid (GA) has been shown to specifically bind to receptors on the liver cell membrane, as there are more glycyrrhetinic acid binding sites in hepatoma cells than in other cells [15]. Therefore, nanomaterials combined with glycyrrhetinic acid will tend to accumulate in hepatoma cells, leading to improved growth inhibition [16,17].

In this study, we prepared glycyrrhetinic acid-modified chitosan (GA-CTS), which was used to synthetize a GA-CTS/5-FU nanoparticle. The results showed that the sustained release system (GA-CTS/5-FU nanoparticle) efficiently targeted the drug to liver and significantly inhibited tumor growth in an orthotropic liver cancer mouse model, resulting in increased survival time.

## 2. Results

### 2.1. Fourier Transformed Infrared Spectroscopy (FT-IR) Spectra and ^1^H-Nuclear Magnetic Resonance (^1^H-NMR) of GA-CTS

**Figure 1:**
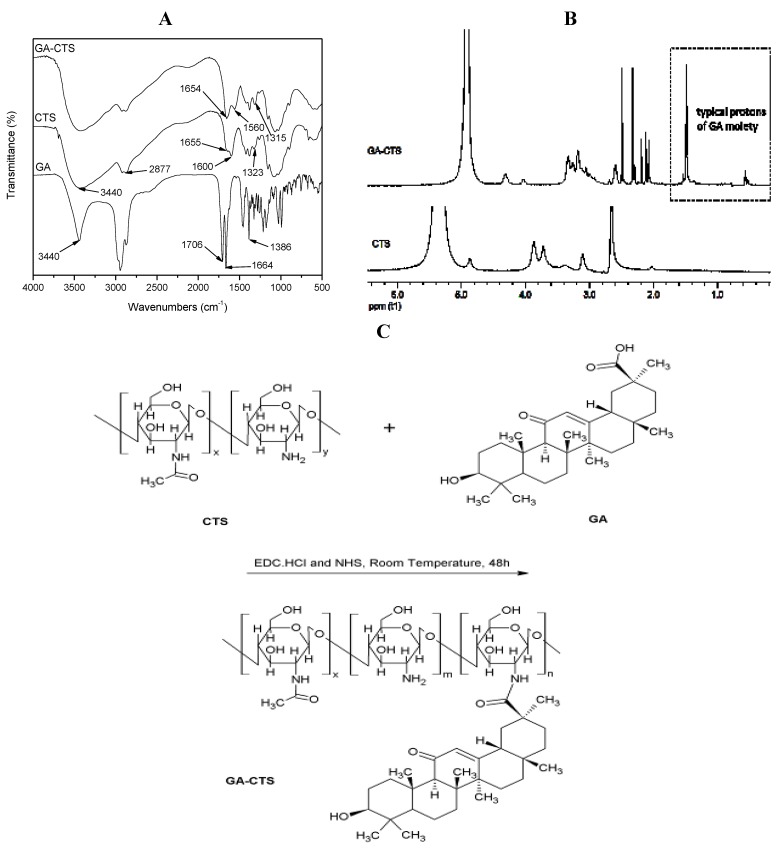
Fourier transformed infrared spectroscopy (FT-IR) spectra and ^1^H-nuclear magnetic resonance (^1^H-NMR) of glycyrrhetinic acid-modified chitosan (GA-CTS). (**A**) The mass spectrum of GA-CTS; the amide bond was formed by the carboxylic acid group of GA and the amino group of chitosan (CTS); (**B**) ^1^H-NMR of GA-CTS showing that GA was connected to CTS; (**C**) Synthetic scheme for GA-CTS.

Figure 1A shows the FT-IR spectra of GA, CTS and GA-CTS. FT-IR spectra of GA existed as an oleanane-type pentacyclic triterpene skeleton. The characteristic absorption peak was at the bending vibration absorption of the C-H in-plane group of the gem-dimethyl moiety connected to C_4_. The characteristic absorption peak of the p-π conjugated carbonyl group of C_11_ was at 1664 cm^−1^. It existed as a C_30_ carboxyl group with an absorption peak at 1706 cm^−1^. The peak at 3440 cm^−1^ represented the stretch vibration absorption peak of C_3_. The FT-IR spectra of CTS showed three characteristic amide absorption peaks: an amide band I at 1655 cm^−1^, an amide band II at 1600 cm^−1^ and an amide band III at 1323 cm^−1^. The relatively weak absorption peak of amide band I and the relatively strong absorption peak of amide band II indicate that there was a high degree of deacetylation of CTS. Characteristic absorption peaks of carbohydrates were observed at 1155 cm^−1^ for the asymmetric stretching vibration absorption peak at C–O–C and at 1078 cm^−1^ and 1025 cm^−1^ for the skeleton stretching vibration absorption peak of C-O. The stretching vibration absorption peak of O-H and N-H was represented by the wave at 3440 cm^−1^ and by the stretching vibration peak at 2877 cm^−1^. Absorption band shifts for GA/CTS were seen at 1655 cm^−1^ of amide band I, 1600 cm^−1^ of amide band II and 1323 cm^−1^ of amide band III in CTS and at 1654 cm^−1^, 1560 cm^−1^ and 1314 cm^−1^, respectively, in the GA/CTS nanoparticles. The intensity of amide band I increased, while the intensity of amide band II decreased. The absorption band of the carboxyl group in GA (1706 cm^−1^) disappeared. These changes were attributed to the formation of the amide bond between the GA carboxyl group and the CTS amine. Figure 1B shows the ^1^H-NMR results of CTS and GA-CTS. Chemical shifts of 1.410 ppm and strong characteristic absorption peaks at 0.566 were observed for CTS, which were attributed to the protons in the -CH_3_, -CH_2_ and -CH groups of GA. These data indicate the successful connection of GA to the amino group of CTS. 

### 2.2. Characterization of GA-CTS/5-FU Nanoparticles

**Figure 2:**
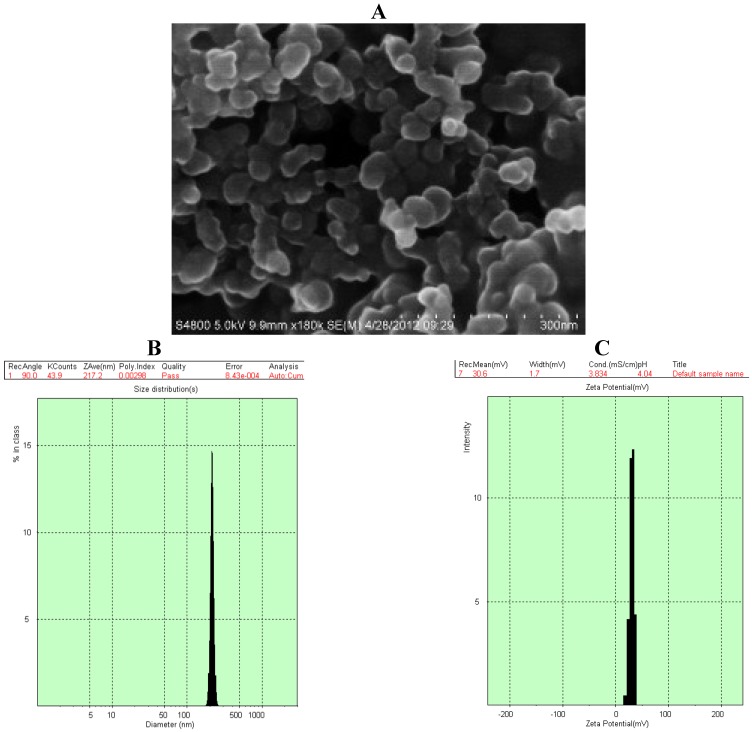
Scanning electron microscopy (SEM) chart and characteristics of GA-CTS/5-fluorouracil (5-FU) nanoparticle. (**A**) The particles were spherical, with a smooth surface, and there was no adhesion between nanoparticles; (**B**) The particle size graph showing the diameter of GA-CTS/5-FU (217.2 nm); (**C**) The particle size graph showing the zeta potential of GA-CTS/5-FU (30.6 mV); (**D**) The *in vitro* release curve of nanoparticles in simulated body fluid (37 °C at pH 7.4). Data are the mean ± SD (*n* = 3).

Scanning electron microscopy (SEM) of GA-CTS/5-FU showed the presence of spherical nanoparticles (sized 217.2 nm) with smooth surfaces (Figure 2A). The polydispersity index (PI) was 0.003, indicating good dispersion (Figure 2B). The zeta potential was +30.6 mV (Figure 2C) and drug loading efficiency was 1.56%.

To study the release of GA-CTS/5-FU nanoparticles, an *in vitro* release curve was generated using simulated body fluid. Linear regression of the peak area ratio of the 5-FU concentration (0.1 to 20 mg/L) curve was calculated as *y* = 2.5721*x* + 0.6851 (*r* = 0.9956). Rapid release was observed for 5-FU in simulated body fluid (SBF) with a cumulative release percentage of 95.7% within 1 h. Nanoparticle release occurred in three stages (Figure 2D). Rapid release was observed between 0 and 6 h, which was followed by a cumulative release of 20.6%, due to the diffusion of surface 5-FU into the SBF solution. A smooth slow-release process occurred between 6 h and day 7, resulting in a cumulative release of 91.4%. During day 7 to 10, the release reached a plateau, with a cumulative release percentage of 95.6% and additional release of only 4.2% on day 10.

### 2.3. Dose- and Time-Dependent Cytotoxicity of GA-CTS Nanoparticles in Tumor Cells

**Figure 3:**
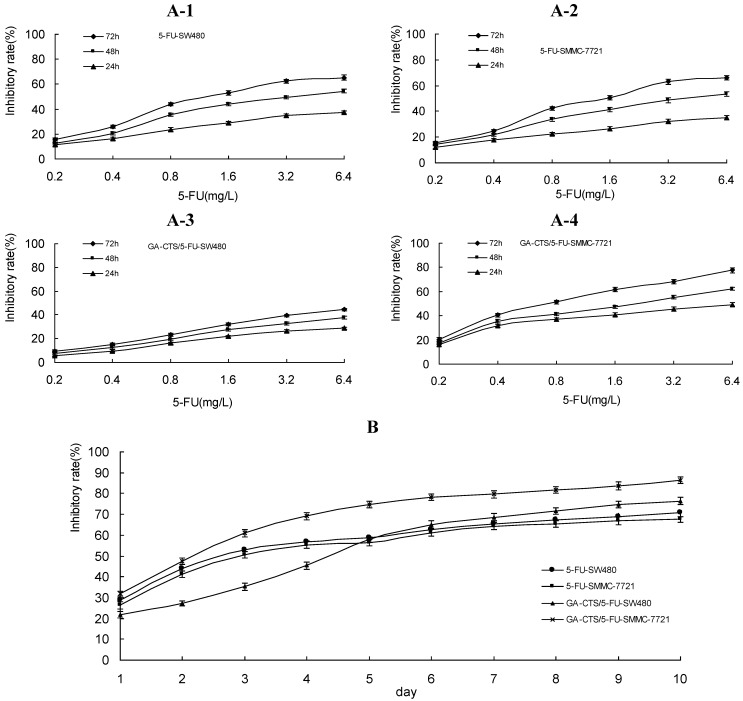
Inhibition rates of GA-CTS/5-FU and 5-FU in SW480 and SMMC-7721 cells detected by methyl thiazolyl tetrazolium (MTT) assays. Data are the mean ± SD (*n* = 3). (**A**) Inhibition rates of different doses of 5-FU on SW480 and SMMC-7721 cells at 24, 48 and 72 h; (**B**) Inhibition rates of different groups on SW480 and SMMC-7721 cells after one to 10 days (5-FU: 1.6 mg/L).

As shown in Figure 3A, GA-CTS/5-FU and 5-FU inhibited the growth of tumor cells in a dose-dependent manner. At 24, 48 and 72 h, tumor growth inhibition rates were the highest for GA-CTS/5-FU in SMMC-7721 cells, followed by 5-FU in SW480 cells, 5-FU in SMMC-7721 cells and GA-CTS/5-FU in SW480 cells, indicating the maximum inhibition of hepatic cancer cells by GA-CTS/5-FU (*p* < 0.01). Between days 1 and 5, the inhibition rate for GA-CTS/5-FU was lower than for 5-FU in SW480 and SMMC-7721 cells. By contrast, the cytotoxic effect of GA-CTS/5-FU was higher days 6 to 10, possibly indicating the release of GA-CTS/5-FU nanoparticles, resulting in a longer duration of action (Figure 3B).

### 2.4. Cytotoxicity of GA-CTS Nanoparticles in Drug-Resistant Hepatoma Cells

**Figure 4:**
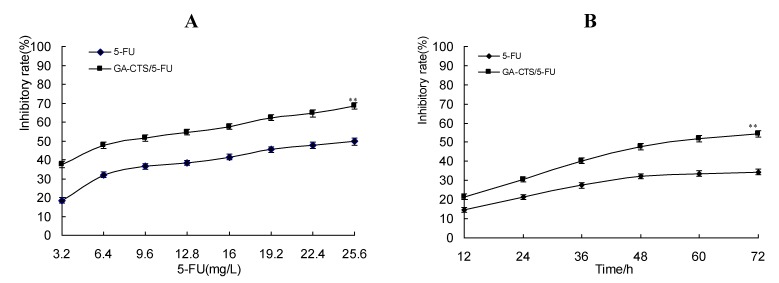
Inhibition of GA-CTS/5-FU and 5-FU drug-resistant SMMC-7721 cells detected by MTT assay. Data are the mean ± SD (*n* = 3). (**A**) Inhibition rates of different doses of drugs on SMMC-7721 cells resistant to 5-FU; (**B**) Inhibition rates of different times on drug-resistant cell lines (5-FU: 6.4 mg/L). ** *p* < 0.01 compared with the 5-FU group.

A moderately drug-resistant hepatoma cell line (resistance index (RI): 8.6) was established to study the inhibitory activity of GA-CTS/5-FU nanoparticles in drug-resistant cells. 50% inhibiting concentration (IC_50_) values for the inhibitory activity of 5-FU on parent and drug-resistant SMMC-7721 cells was 6.76 mg/L and 32.34 mg/L, respectively, while the IC_50_ of GA-CTS/5-FU on parent and drug-resistant SMMC-7721 cells was 2.05 mg/L and 8.33 mg/L; its RI was 4.06 (Table 1). As shown in Figure 4A, the cytotoxicity of drugs on the drug-resistant cell lines was dose-dependent. Inhibition was significantly higher with GA-CTS/5-FU than with 5-FU at the same concentration (*p* < 0.01). All groups inhibited tumor growth in a time-dependent manner. Cytotoxicity in the GA-CTS/5-FU group was significantly higher than in the 5-FU group (Figure 4B, *p* < 0.01). Taken together, these results indicate a strong inhibitory effect of GA-CTS/5-FU in drug-resistant hepatoma cells.

**Table 1:** Inhibitory effects of 5-FU and GA-CTS/5-FU on proliferation of the SMMC-7721 parental cell line and the SMMC-7721 5-FU resistance cell line (mean ± SD, *n* = 3).

Parental cell line of SMMC-7721				5-FU resistance SMMC-7721 cell line			
Concentrations (mg/L)	A Value	Inhibitory rate (%)	IC_50_ (mg/L)	Concentrations (mg/L)	A Value	Inhibitory rate (%)	IC_50_ (mg/L)
5-FU							
0	0.65 ± 0.06			0	0.83 ± 0.06		
0.2	0.55 ± 0.05	14.64		3.2	0.67 ± 0.05	18.34	
0.4	0.51 ± 0.07	21.65		6.4	0.56 ± 0.06	32.10	
0.8	0.43 ± 0.04	33.65	3.76	12.8	0.51 ± 0.05	38.24	32.34
1.6	0.38 ± 0.03	41.25		25.6	0.42 ± 0.04	49.60	
3.2	0.33 ± 0.03	48.65		51.2	0.39 ± 0.04	53.21	
6.4	0.30 ± 0.04	53.64		102.4	0.29 ± 0.03	65.40	
GA-CTS/5-FU							
0	0.63 ± 0.05			0	0.81 ± 0.04		
0.2	0.52 ± 0.04	17.35		3.2	0.51 ± 0.05	37.45	
0.4	0.41 ± 0.04	35.46		6.4	0.42 ± 0.04	47.74	
0.8	0.37 ± 0.03	41.52	2.05	9.6	0.39 ± 0.03	51.35	8.33
1.6	0.33 ± 0.03	47.35		12.8	0.37 ± 0.04	54.58	
3.2	0.28 ± 0.02	55.35		16.0	0.34 ± 0.03	57.65	
6.4	0.24 ± 0.02	62.18		19.2	0.31 ± 0.03	62.24	

### 2.5. Determination of *in Vitro* and *in Vivo* Liver Targeting of GA-CTS/5-FU

**Figure 5:**
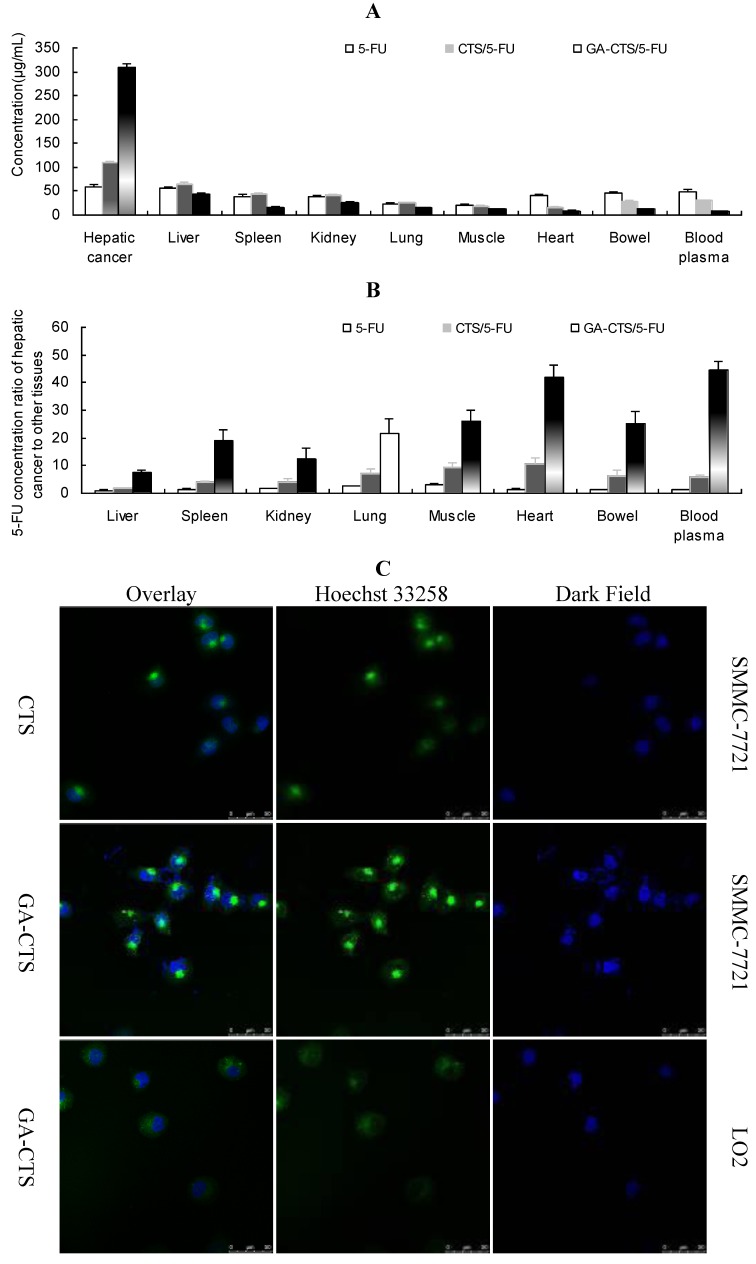
GA-CTS/5-FU liver targeting *in vitro* and *in vivo*. (**A**) Mice with experimentally-induced orthotropic liver cancer were treated with 5-FU, CTS/5-FU and GA-CTS/5-FU administered via the tail vein by injection. Mice were sacrificed after 30 min; 5-FU concentrations in different tissues were evaluated. Data are the mean ± SD (*n* = 3); (**B**) 5-FU concentrations in hepatic cancer, liver, spleen, kidney, lung, muscle, heart and small intestine and blood; (**C**) Fluorescence images of SMMC-7721 and LO2 cells incubated with fluorescein isothiocyanate (FITC)-labeled GA-CTS for 4 h (*n* = 3).

We used an orthotropic liver cancer mouse model to determine the *in vivo* targeting of GA-CTS/5-FU. Concentrations of 5-FU were measured in different tissues 30 min after injection of 5-FU, CTS/5-FU and GA-CTS/5-FU. Significant differences were observed for the concentrations of 5-FU after each treatment (Figure 5A, *p* < 0.01). The concentration of 5-FU in hepatic cancer cells, after treatment with GA-CTS/5-FU, was, respectively, 2.81- and 5.81-times higher than in mice treated with CTS/5-FU or 5-FU. The lowest concentration was seen with 5-FU. In other tissues, the levels of 5-FU were lower following treatment with GA-CTS/5-FU than in the other groups. Following administration of GA-CTS/5-FU, the concentration of 5-FU in hepatic cancer cells was 7.29-, 12.26-, 41.99- and 44.71-times higher than in normal liver, kidney, heart and blood, respectively (Figure 5B). These results indicate targeting of GA-CTS in liver.

Endocytosis of GA-CTS nanoparticles by normal liver LO2 cells and by hepatic cancer SMMC-7721 cells was analyzed under confocal microscopy. As shown in Figure 5C, strong green fluorescence was observed in SMMC-7721 cells following exposure to GA-CTS nanoparticles, indicating endocytosis of a large number of GA-CTS particles. SMMC-7721 cells exposed to CTS nanoparticles showed moderate intensity fluorescence. However, only weak intensity fluorescence was observed in LO2 cells exposed to GA-CTS nanoparticles. These data indicate that GA-CTS nanoparticles target hepatic cancer cells and entered the cells through receptor-mediated endocytosis.

### 2.6. *In Vivo* Anti-Tumor Efficacy of GA-CTS/5-FU

**Figure 6:**
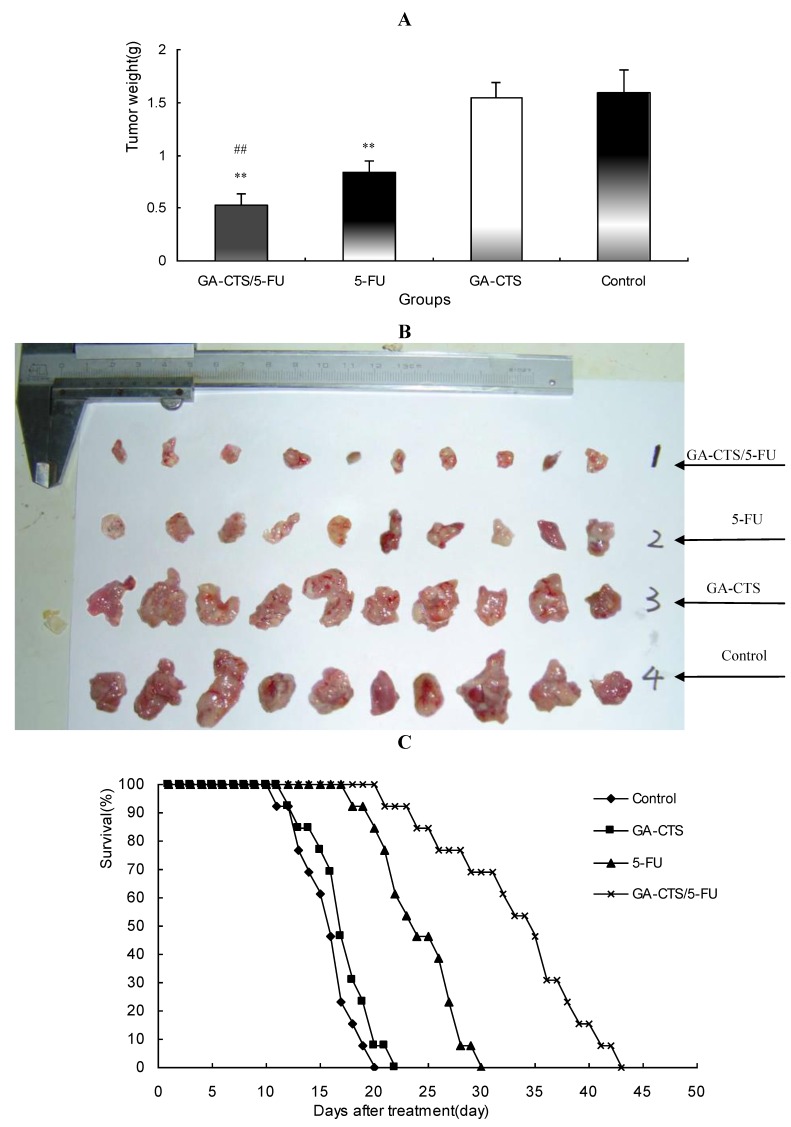
The effect of GA-CTS/5-FU on an orthotopic cancer liver model in mice. (**A**) On day 5, mice were treated with GA-CTS/5-FU, 5-FU, GA-CTS and phosphate buffered solution (PBS). After 10 days, the mice were sacrificed and the tumors were weighed. Results are the mean ± SD (*n* = 10); (**B**) The general picture of tumor tissue; (**C**) Kaplan-Meier analysis indicated that median survival was highest for GA-CTS/5-FU, followed by 5-FU, GA-CTS and the control group (*n* = 13). ** *p* < 0.01 compared with the control or GA-CTS; ## *p* < 0.01 compared with 5-FU.

As shown in Figure 6A,B, tumor weights were significantly lower in the GA-CTS/5-FU group (0.52 ± 0.11 g) than in the GA-CTS (1.62 ± 0.15 g) or control groups (1.64 ± 0.21 g; *p* < 0.01). In the 5-FU group, tumor weight was significantly less than in the control group (*p* < 0.01), but no differences were seen between the GA-CTS group and controls (*p* > 0.05).

Kaplan-Meier analysis showed that the median survival was 16 days in the control group, with all the mice dying between day 11 and 20 (Figure 6C). Mice in the GA-CTS group had a median survival of 17 days, with all the mice dying between day 12 and 22. Mice died between day 18 and 30 in the 5-FU group, with a median survival of 24 days. Mice treated with GA-CTS/5-FU had a significantly longer median survival (35 days) than in other groups (*p* < 0.01), with all mice dying between 21 and 43 days.

## 3. Discussion

Targeted drug therapy can specifically deliver drugs to tumor tissues, resulting in increased local concentrations and reduced toxicity. Glycyrrhetinic acid, an aglycone of glycyrrhizin, acts as an antioxidant and detoxifying agent. It has been shown to increase apoptosis in hepatoma cells [18,19]. It also has the ability to target the liver [20,21] and has been shown to specifically bind to receptors on the liver cell membrane [22]. These properties make glycyrrhetinic acid a suitable candidate for use in the development of novel drugs that target liver disease (Figure 1C).

In the present study, GA-CTS identified by IR and ^1^H-NMR was used to investigate the feasibility of using GA-CTS as a drug carrier to the liver. 5-FU is a widely used anti-cancer drug, which is toxic to normal cells and has a short plasma half-life of 15 to 20 min. Improving the bioavailability and site-specific delivery of 5-FU and reducing its side effects may provide obvious therapeutic advantages. In this study, we prepared GA-CTS/5-FU nanoparticles with an average diameter of 217.2 nm. SEM imaging showed that GA-CTS/5-FU nanoparticles were spherical, smooth surface structures that adhered to one another and were not fully dispersed after centrifugation. The polydispersity index (PI) is an important indicator of the physical stability of nanoparticles. PI values between 0.1 and 0.25 indicate good uniformity, while values >0.5 are indicative of poor uniformity [23]. Prior to centrifugation, the GA-CTS/5-FU nanoparticles used in our study had PI value of 0.003, with a single peak, indicating good uniformity. The release of nanoparticles in SBF occurred in stages, characterized by sustained release, which was similar to previous studies [24]. Rapid release was observed between zero to 6 h, with a cumulative release of 20.6%, resulting from diffusion of surface 5-FU into the SBF solution. Smooth, slow-release occurred after between 6 h and 7 days. Following the gradual degradation of insoluble, hydrophobic material, the drug diffused through the membrane, resulting in a cumulative release of 60.8% between day 7 and 10 and a residual drug release of 4.2% on day 10.

The anti-tumor mechanism of 5-FU is associated with the metabolite, 5-F-deoxy-uridine monophosphate (dUMP), which inhibits thymidylate synthase (TS) activity and blocks the methylation of dUMP to deoxythymidine monophosphate (dTMP) which, in turn, affects DNA synthesis [25,26]. Between day 1 and 5, the inhibition for CTS/5-FU was lower than 5-FU, probably due to the slow release of 5-FU encapsulated by CTS and low cellular concentrations 5-FU. The inhibition reached a plateau between day 6 and 10. This profile was not observed with the GA-CTS nanoparticle group, which had a longer duration of action. These findings are in general agreement with our previous report demonstrating the inhibition of tumor cells by galactosyl chitosan/5-FU [27].

To investigate the effect of GA-CTS/5-FU nanoparticles on drug-resistant cells, we established a SMMC-7721 cell line that was moderately resistant to 5-FU (RI: 8.6) [28]. Our results demonstrated that GA-CTS/5-FU significantly inhibited the proliferation of SMMC-7721 cells that were resistant to 5-FU. Cytotoxicity in the GA-CTS/5-FU group was significantly higher than in the 5-FU group. 5-FU is both poorly fat soluble and poorly water soluble. The mechanism of drug resistance might therefore be related to membrane, enzyme and genetic abnormalities [29,30,31]. GA-CTS nanoparticles enter cells by binding to GA receptors on the membrane. This is followed by a process of endocytosis, which is different from the mechanism seen with 5-FU, making the particles effective against drug-resistant cells.

To explore the liver targeting efficiency of GA-CTS/5-FU nanoparticles, *in vivo* distribution was evaluated. Absorption in hepatoma cells was analyzed using confocal microscopy. The results showed that the concentration of 5-FU in hepatocellular carcinoma (HCC) from GA-CTS/5-FU was 2.81-, 5.81- and 7.29-times higher than that achieved with CTS/5-FU, 5-FU and the control, respectively. Confocal microscopy provided evidence of a stronger green fluorescence in hepatoma cells exposed to GA-CTS nanoparticles than in those exposed to CTS. Hepatoma cells exposed to GA-CTS nanoparticles also had a stronger green fluorescence than normal liver cells. These results provide evidence of the enrichment of GA-CTS nanoparticles on the surface of hepatoma cells, which facilitates the binding of GA binding to GA receptors or other binding sites. This is followed by endocytosis of 5-FU at optimal local concentrations [16].

Our results also demonstrated that tumor weight in a mouse orthotopic liver transplantation model was significantly lower in animals who had received GA-CTS/5-FU than in other groups. No antitumor effect was observed in the GA-CTS group. Kaplan-Meier analysis showed longer median survival in the GA-CTS/5-FU than in 5-FU group, further indicating better anti-tumor efficacy. This suggests that GA binding might result in a higher concentration of 5-FU in the hepatoma cells [32]. GA-CTS/5-FU also attenuated the toxicity caused by 5-FU.

## 4. Experimental Section

### 4.1. Mice and Cell Lines

The human hepatocellular carcinoma cell line (SMMC-7721) and normal liver cells (LO2) were obtained from the Committee on Type Culture Collection of the Chinese Academy of Sciences (Shanghai, China). The human colon cancer cell line (SW480) was purchased from the American Type Culture Collection (Manassas, VA, USA), and the mouse hepatoma cell line, H22, was purchased from the China Center for Type Culture Collection (CCTCC, Wuhan, China). 

Female BALB/c mice, 7 weeks of age, weighing about 20 g, were obtained from the Science Department of Experimental Animals of Fudan University in China and kept under standard conditions of an animal room (temperature, 25 °C; humidity, 55%~60%) in a specific pathogen-free level B animal facility. All animal care and experimental protocols complied with the Animal Management Rules of the Ministry of Health of the People’s Republic of China and were approved by the ethics committee of Shanghai Zhoupu Hospital (Shanghai, China) and Fudan University.

### 4.2. GA-CTS Synthesis

EDC.HCl (1-ethyl-3-(3-dimethyl aminopropyl) carbodiimide hydrochloride, Sigma, St. Louis, MO, USA) and NHS (N-hydroxysuccinimide, Sigma, St. Louis, MO, USA) were added to GA solution (1 g, Xi’an Fujie Pharmaceutical Co., Ltd, Yilu, China) in DMF (dimethylformamide, Amresco, Solon, OH, USA). The solution was mixed with 2% chitosan in acetic acid and stirred at room temperature. After 48 h, the mixture was precipitated with acetone, and the precipitate was washed with 60% ethanol and ether. The final product was obtained after vacuum drying.

### 4.3. FT-IR Spectroscopy

Eighty-five percent deacetylated CTS powder (Sigma, St. Louis, MO, USA) and GA-CTS were pressed into a potassium bromide (KBr) pellet. The composites were analyzed by FT-IR (NEXUS, Nicolet, USA) in the range 400–4000 cm^−1^.

### 4.4. ^1^H-NMR Experiments

To verify the structure of CTS and GA-CTS, samples were dissolved in a solution of deuterium chloride and D_2_O. ^1^H-NMR spectra were recorded using a Varian NMR System 600 machine at a resonance frequency of 600 MHz. Tetramethylsilane (TMS) was used as a reference compound.

### 4.5. Preparation of GA-CTS/5-FU Nanoparticles

GA-CTS/5-FU nanoparticles were prepared by the ionic crosslinking method. Briefly, 32 mg of GA-CTS was dissolved in 8 mL aqueous solution of acetic acid (1% by w/v), and the pH was adjusted to 4.7 with 0.1 M NaOH. Subsequently, 32 mg of 5-FU was incorporated in the above solution. Next, 12.8 mL 0.05% (w/v) sodium polyphosphate (TPP) solution was added dropwise into the above solution under magnetic stirring at a low velocity and allowed to crosslink for 50 min. The formation of nanoparticles started spontaneously via the TPP initiated ionic gelation mechanism. The suspension was subjected to repeated cycles of washing by deionized water and centrifugation (10,000 rpm for 30 min) to remove the unreacted CTS, TPP and NaOH to separate nanoparticles, then lyophilized and stored at 4 °C.

Particle size distribution and zeta potential were determined by dynamic light scattering and a laser particle size analyzer (Zetasizer-3000HS, Malvern Instruments Ltd., Worcestershire, UK) equipped with a 10 mW helium-neon laser at a wavelength of 633 nm, 90° scattering angle and temperature of 25 °C. The suspension liquid of drug-loaded nanoparticles was prepared in the cuvette. The size and distribution of nanoparticles were measured by putting the cuvette into the sample cell. The zeta potential of the nanoparticle suspension was measured in the pipeline, which was put into a 5 mL syringe. The nanoparticles were centrifuged at 10,000 rpm for 30 min to remove NaOH and 5-FU and were dispersed in ddH_2_O; these procedures were repeated three times. The suspension liquid of nanoparticles was dispersed and dropped onto foil, after drying in an oven. The morphology of the nanoparticles was observed by a field emission scanning electron microscope (FE-SEM, Hitachi S-4800, Hitachi Ltd., Tokyo, Japan).

### 4.6. *In Vitro* Release of GA-CTS/5-FU Nanoparticles

To prepare a standard curve, a series of working solutions of 5-FU (0.1, 0.2, 0.5, 1.0, 5.0, 10, 20 mg/L) were obtained by diluting 5-FU standard stock solution with 0.5 mL human plasma. A calibration curve was generated by linear regression of the peak area ratio of the 5-FU concentration.

GA-CTS/5-FU nanoparticles and 5-FU were placed into dialysis bags and immersed in 30 mL of SBF at pH 7.4. The particles were incubated at 37 °C and shaken at a constant speed of 60 rpm/min. The medium was replaced with fresh SBF at 20 and 40 min, 1 and 6 h and, then, daily for 10 days. Drug concentration in each sample was assayed in triplicate by measuring the absorbance at 265 nm. Drug release and loading capacity were calculated by the following formulas:

Cumulative drug release (%) = (release of 5-FU from samples)/(total 5-FU) × 100%

Drug loading (%) = (mass of 5-FU encapsulated in nanoparticles)/(mass of nanoparticles) ×100%

### 4.7. Cytotoxicity of GA-CTS/5-FU Nanoparticles

SW480 and SMMC-7721 cells were grown in roosevelt park memorial institute (RPMI) 1640 medium with 10% fetal calf serum at 37 °C in 5% CO_2_. A cell suspension containing 1 × 10^4^ cells was plated into 96-well plates at concentrations of 190 μL/well for 24 h. Cells were exposed to 10 μL of 5-FU or GA-CTS/5-FU nanoparticles at different doses for 24, 48, 72 h. MTT (30 µg; Sigma Ltd., Shanghai, China) was added to each well and incubated for 4 h. The medium was then removed, 200 μL of DMSO was added and the plates were vibrated for 10 min. Absorbance was measured by a Bio-Rad automatic micro-plate reader at 490 nm. All measurements were performed in triplicate. MTT assays were performed after exposure for 4 to 10 days with 1.6 mg/L of 5-FU.

Cytotoxicity was determined as follows:

Cytotoxicity (%) = (A_490_ of control − A_490_ of sample)/A_490_ of control × 100%

### 4.8. Establishment of SMMC-7721/5-FU Drug-Resistant Cell Lines and Calculation of Resistance Index

A drug-resistant SMMC-7721/5-FU cell line was established using concentration gradients and increased induction [33]. Briefly, 20 mL of SMMC-7721 cells (1 × 10^4^/mL) in logarithmic phase growth were cultured for 24 h at 37 °C in 5% CO_2_ in 100 mL culture flasks containing RPMI 1640 medium supplemented with 10% neonatal bovine serum (NBS). At the end of the incubation period, the culture solution was replaced with 5-FU (3.2 mg/mL). Forty-eight hours later, the solution containing the drugs was discarded and replaced with 5-FU solution at (0.8 mg/mL). The process was repeated three times, such that the cells were successively incubated with 5-FU at 1.6, 6.4 and 12.8 mg/mL. After two months, cells in logarithmic phase growth were cultured in medium containing 3.2 mg/mL 5-FU.

SMMC-7721/5-FU drug-resistant cells and SMMC-7721 cells (200 μL) were plated into 96-well plates at a concentration of 1 × 10^4^/mL. After incubation for 24 h, the cells were centrifuged and collected. SMMC-7721/5-FU drug-resistant cells were re-inoculated with different doses of 5-FU (0, 3.2, 6.4, 12.8, 25.6, 51.2 and 102.4 mg/L, *n* = 3) and GA-CTS/5-FU (0, 3.2, 9.6, 12.8, 16.0 and 19.2 mg/L, *n* = 3). SMMC-7721 cells were incubated with lower concentrations of 5-FU and GA-CTS/5-FU (0, 0.2, 0.4, 0.8, 1.6, 3.2 and 6.4 mg/L, *n* = 3). Absorbance at 490 nm was measured at 48 h using the MTT method. IC_50_ values and cytotoxicity were determined. The resistance index (RI) was estimated using the equation:

Resistance index (RI) = IC_50_ of drug − resistant cells/IC_50_ value of parent cells

### 4.9. Inhibition of GA-CTS/5-FU Nanoparticles on SMMC-7721/5-FU Drug-Resistant Cells

SMMC-7721/5-FU cells (200 μL) were plated into 96-well plates at a concentration of 1 × 10^4^/mL. After incubation for 24 h, cells were centrifuged, collected and incubated with 5-FU (3.2, 6.4, 12.8, 16, 19.2, 22.4 and 25.6 mg/L) or the same doses of GA-CTS/5-FU nanoparticles for 48 h. Two additional groups of cells were incubated with 6.4 mg/mL of 5-FU and GA-CTS/5-FU nanoparticles and were cultured for 12, 24, 36, 48, 60 and 72 h. MTT assays were performed to determine the cytotoxicity of the hepatocellular carcinoma cells.

### 4.10. Orthotopic Transplant Mouse Model of Hepatocellular Carcinoma

Mice with cancer were sacrificed, the tumors were extracted and a cell suspension containing 6 × 10^7^ cells was prepared. Recipient mice were anesthetized by intraperitoneal injection of 20% urethane. Then, 50 µL of cell suspension were subcutaneously implanted in the left lobe of the liver. The abdomen was closed after 2 min.

### 4.11. Targeting GA-CTS/5-FU Nanoparticles in Hepatocellular Carcinoma *In Vivo*

Following tumor implantation, the mice were randomly divided into three groups (*n* = 5) and treated with 200 µL of 5-FU, CTS/5-FU or GA-CTS/5-FU (5-FU: 0.371mg). The mice were sacrificed after 30 min. Liver tumor, liver, spleen, kidney, lung, muscle, heart and small intestine were harvested, washed in saline solution and dried on filter papers. The tissue (0.5–1.0 g) was homogenized, and the concentration of 5-FU in 0.5 mL of the homogenates was determined.

### 4.12. Targeting of GA-CTS Nanoparticles in Hepatic Cancer *in Vitro*

SMMC-7721 and LO2 cells were plated into 6-well plates and incubated for 24 h. The cells were exposed to fresh medium containing CTS or GA-CTS nanoparticles labeled with different FITC concentrations for 4 h. The cells were fixed in 4% paraformaldehyde at room temperature for 20 min and stained with Hoechst 33258 nuclear dye (Sigma, St. Louis, MO, USA). After washing three times in 0.01 M PBS, coverslips were added, and the slides were observed under a fluorescence microscope using a 488 nm excitation filter for FITC and a 405 nm filter for Hoechst 33258. The images were analyzed using NIS-Elements software.

### 4.13. Inhibitory Effects of GA-CTS/5-FU Nanoparticles in and Orthotopic Liver Transplantation Mouse Model

Five days after establishment of the orthotopic liver transplantation mouse model, tumor size reached 4–6 mm. Six days after model establishment, mice were divided into four groups: control, GA-CTS, 5-FU and GA-CTS/5-FU. Mice in the four groups were injected with saline, GA-CTS, 5-FU or GA-CTS /5-FU nanoparticles, respectively, at a volume of 200 µL (all 5-FU-containing injections contained 0.371 mg of 5-FU). Drugs were administered for 5 days, and 10 mice in each group were killed at 10 days by an overdose of anesthesia to harvest their tumor tissues. All efforts were made to ameliorate animal suffering. The tumor growth and tumor weight were measured in each group. The remaining 13 mice in each group were used for survival analysis.

### 4.14. Statistical Analysis

All data were expressed as the means and standard deviations (±SD). One-way analysis of variance (ANOVA) and the least significance difference (LSD) test were used for intergroup comparisons. Kaplan-Meier survival plots were used to analyze survival data. In all analyses, values of *p* < 0.05 were considered statistically significant.

## 5. Conclusions

In this study, we successfully synthesized a GA-CTS/5-FU nanoparticle as a sustained release system, which had a dose- and time-dependent anti-cancer effect and may compensate for the drug resistance of 5-FU. GA-CTS/5-FU nanoparticle significantly inhibited tumor growth against orthotropic liver cancer in mice, resulting in increased survival time.

## References

[1] Toriumi F., Kubota T., Saikawa Y., Yoshida M., Otani Y., Watanabe M., Kumai K., Kitajima M. (2004). Thymidylate synthetase (TS) genotype and TS/dihydropyrimidine dehydrogenase mRNA level as an indicator in determining chemosensitivity to 5-fluorouracil in advanced gastric carcinoma. Anticancer Res..

[2] Johnson K.R., Wang L., Miller M.R., Willingham M.C., Fan W. (1997). 5-Fluorouracil interferes with paclitaxel cytotoxicity against human solid tumor cells. Clin. Cancer Res..

[3] Fang J.Y., Liu P.F., Huang C.M. (2008). Decreasing systemic toxicity via transdermal delivery of anticancer drugs. Curr. Drug Metab..

[4] Chen Y., Wang X., Yan Z., Wang J., Luo J., Liu Q. (2012). Hepatic arterial infusion with irinotecan, oxaliplatin, and floxuridine plus systemic chemotherapy as first-line treatment of unresectable liver metastases from colorectal cancer. Onkologie.

[5] Takahashi Y., Yamashita K., Endo Y., Sasaki T., Mai M. (2004). Oral administration of uracil-tegafur (UFT) inhibits liver micrometastasis of human colon cancer in an orthotopic nude mouse model and its early detection system. Surg. Today.

[6] Oh I.H., Min H.S., Li L., Tran T.H., Lee Y.K., Kwon I.C., Choi K., Kim K., Huh K.M. (2013). Cancer cell-specific photoactivity of pheophorbide a-glycol chitosan nanoparticles for photodynamic therapy in tumor-bearing mice. Biomaterials.

[7] Hamman J.H. (2010). Chitosan based polyelectrolyte complexes as potential carrier materials in drug delivery systems. Mar. Drugs.

[8] Arulmozhi V., Pandian K., Mirunalini S. (2013). Ellagic acid encapsulated chitosan nanoparticles for drug delivery system in human oral cancer cell line (KB). Colloids Surf. B Biointerfaces.

[9] Javid A., Ahmadian S., Saboury A.A., Kalantar S.M., Rezaei-Zarchi S. (2013). Chitosan Coated Superparamagnetic Iron Oxide Nanoparticles for Doxorubicin Delivery: Synthesis and Anticancer Effect against Human Ovarian Cancer Cells. Chem. Biol. Drug Des..

[10] Huang X., Wang Y., Cai J.P., Ma X.Y., Li Y., Cheng J.W., Wei R.L. (2013). Sustained release of 5-fluorouracil from chitosan nanoparticles surface modified intra ocular lens to prevent posterior capsule opacification: an *in vitro* and *in vivo* study. J. Ocul. Pharmacol. Ther..

[11] Xu J., Ma L., Liu Y., Xu F., Nie J., Ma G. (2012). Design and characterization of antitumor drug paclitaxel-loaded chitosan nanoparticles by W/O emulsions. Int. J. Biol. Macromol..

[12] Yan S., Zhu J., Wang Z., Yin J., Zheng Y., Chen X. (2011). Layer-by-layer assembly of poly(*L*-glutamic acid)/chitosan microcapsules for high loading and sustained release of 5-fluorouracil. Eur. J. Pharm. Biopharm..

[13] Park J.H., Saravanakumar G., Kim K., Kwon I.C. (2010). Targeted delivery of low molecular drugs using chitosan and its derivatives. Adv. Drug Deliv. Rev..

[14] Yu J.M., Li Y.J., Qiu L.Y., Jin Y. (2009). Polymeric nanoparticles of cholesterol-modified glycol chitosan for doxorubicin delivery: Preparation and *in vitro* and *in vivo* characterization. J. Pharm. Pharmacol..

[15] He Z.Y., Zheng X., Wu X.H., Song X.R., He G., Wu W.F., Yu S., Mao S.J., Wei Y.Q. (2010). Development of glycyrrhetinic acid-modified stealth cationic liposomes for gene delivery. Int. J. Pharm..

[16] Tian Q., Zhang C.N., Wang X.H., Wang W., Huang W., Cha R.T., Wang C.H., Yuan Z., Liu M., Wan H.Y. (2010). Glycyrrhetinic acid-modified chitosan/poly(ethylene glycol) nanoparticles for liver-targeted delivery. Biomaterials.

[17] Huang W., Wang W., Wang P., Tian Q., Zhang C., Wang C., Yuan Z., Liu M., Wan H., Tang H. (2010). Glycyrrhetinic acid-modified poly(ethylene glycol)-b-poly(gamma-benzyl l-glutamate) micelles for liver targeting therapy. Acta Biomater..

[18] Jeong H.G., You H.J., Park S.J., Moon A.R., Chung Y.C., Kang S.K., Chun H.K. (2002). Hepatoprotective effects of 18beta-glycyrrhetinic acid on carbon tetrachloride-induced liver injury: inhibition of cytochrome P450 2E1 expression. Pharmacol. Res..

[19] Lallemand B., Gelbcke M., Dubois J., Prevost M., Jabin I., Kiss R. (2011). Structure-activity relationship analyses of glycyrrhetinic acid derivatives as anticancer agents. Mini Rev. Med. Chem..

[20] Tian Q., Wang X.H., Wang W., Zhang C.N., Wang P., Yuan Z. (2012). Self-assembly and liver targeting of sulfated chitosan nanoparticles functionalized with glycyrrhetinic acid. Nanomedicine.

[21] Huang W., Wang W., Wang P., Zhang C.N., Tian Q., Zhang Y., Wang X.H., Cha R.T., Wang C.H., Yuan Z. (2011). Glycyrrhetinic acid-functionalized degradable micelles as liver-targeted drug carrier. J. Mater. Sci. Mater. Med..

[22] Negishi M., Irie A., Nagata N., Ichikawa A. (1991). Specific binding of glycyrrhetinic acid to the rat liver membrane. Biochim. Biophys. Acta.

[23] Patravale V.B., Date A.A., Kulkarni R.M. (2004). Nanosuspensions: A promising drug delivery strategy. J. Pharm. Pharmacol..

[24] Zhu L., Ma J., Jia N., Zhao Y., Shen H. (2009). Chitosan-coated magnetic nanoparticles as carriers of 5-fluorouracil: Preparation, characterization and cytotoxicity studies. Colloids Surf. B Biointerfaces.

[25] Ligabue A., Marverti G., Liebl U., Myllykallio H. (2012). Transcriptional activation and cell cycle block are the keys for 5-fluorouracil induced up-regulation of human thymidylate synthase expression. PLoS One.

[26] Nabeya Y., Suzuki T., Furuya A., Koide N., Ohkoshi M., Takiguchi M., Ochiai T., Matsubara H., Hiwasa T. (2011). Calpain regulates thymidylate synthase-5-fluoro-dUMP complex levels associated with response to 5-fluorouracil in gastric cancer cells. Cancer Sci..

[27] Cheng M., He B., Wan T., Zhu W., Han J., Zha B., Chen H., Yang F., Li Q., Wang W. (2012). 5-Fluorouracil nanoparticles inhibit hepatocellular carcinoma via activation of the p53 pathway in the orthotopic transplant mouse model. PLoS One.

[28] Snow K., Judd W. (1991). Characterisation of adriamycin- and amsacrine-resistant human leukaemic T cell lines. Br. J. Cancer.

[29] Kuo M.T., Bao J.J., Curley S.A., Ikeguchi M., Johnston D.A., Ishikawa T. (1996). Frequent coordinated overexpression of the MRP/GS-X pump and gamma-glutamylcysteine synthetase genes in human colorectal cancers. Cancer Res..

[30] Banerjee D., Mayer-Kuckuk P., Capiaux G., Budak-Alpdogan T., Gorlick R., Bertino J.R. (2002). Novel aspects of resistance to drugs targeted to dihydrofolate reductase and thymidylate synthase. Biochim. Biophys. Acta.

[31] Violette S., Poulain L., Dussaulx E., Pepin D., Faussat A.M., Chambaz J., Lacorte J.M., Staedel C., Lesuffleur T. (2002). Resistance of colon cancer cells to long-term 5-fluorouracil exposure is correlated to the relative level of Bcl-2 and Bcl-X(L) in addition to Bax and p53 status. Int. J. Cancer.

[32] Hibasami H., Iwase H., Yoshioka K., Takahashi H. (2006). Glycyrrhetic acid (a metabolic substance and aglycon of glycyrrhizin) induces apoptosis in human hepatoma, promyelotic leukemia and stomach cancer cells. Int. J. Mol. Med..

[33] Gianni M., Koken M.H., Chelbi-Alix M.K., Benoit G., Lanotte M., Chen Z., de The H. (1998). Combined arsenic and retinoic acid treatment enhances differentiation and apoptosis in arsenic-resistant NB4 cells. Blood.

